# Uplift, climate and biotic changes at the Eocene–Oligocene transition in south-eastern Tibet

**DOI:** 10.1093/nsr/nwy062

**Published:** 2018-06-12

**Authors:** Tao Su, Robert A Spicer, Shi-Hu Li, He Xu, Jian Huang, Sarah Sherlock, Yong-Jiang Huang, Shu-Feng Li, Li Wang, Lin-Bo Jia, Wei-Yu-Dong Deng, Jia Liu, Cheng-Long Deng, Shi-Tao Zhang, Paul J Valdes, Zhe-Kun Zhou

**Affiliations:** 1 Key Laboratory of Tropical Forest Ecology, Xishuangbanna Tropical Botanical Garden, Chinese Academy of Sciences, Mengla 666303, China; 2 Key Laboratory for Plant Diversity and Biogeography of East Asia, Kunming Institute of Botany, Chinese Academy of Sciences, Kunming 650204, China; 3 University of Chinese Academy of Sciences, Beijing 100049, China; 4 State Key Laboratory of Paleobiology and Stratigraphy, Nanjing Institute of Geology and Paleontology, Chinese Academy of Sciences, Nanjing 210008, China; 5 School of Environment, Earth and Ecosystem Sciences, The Open University, MK7 6AA, UK; 6 Guangdong Provincial Key Laboratory of Geodynamics and Geohazards, School of Earth Sciences and Engineering, Sun Yat-sen University, Guangzhou 510275, China; 7 Institute of Geology and Paleontology, Linyi University, Linyi 276000, China; 8 State Key Laboratory of Lithospheric Evolution, Institute of Geology and Geophysics, Chinese Academy of Sciences, Beijing 100029, China; 9 Faculty of Land Resource Engineering, Kunming University of Science and Technology, Kunming 650093, China; 10 School of Geographical Sciences and Cabot Institute, University of Bristol, Bristol, BS8 1TH, UK

**Keywords:** biodiversity, Eocene, Oligocene, plant fossil, Qinghai-Tibetan Plateau, uplift

## Abstract

The uplift history of south-eastern Tibet is crucial to understanding processes driving the tectonic evolution of the Tibetan Plateau and surrounding areas. Underpinning existing palaeoaltimetric studies has been regional mapping based in large part on biostratigraphy that assumes a Neogene modernization of the highly diverse, but threatened, Asian biota. Here, with new radiometric dating and newly collected plant-fossil archives, we quantify the surface height of part of the south-eastern margin of Tibet in the latest Eocene (∼34 Ma) to be ∼3 km and rising, possibly attaining its present elevation (3.9 km) in the early Oligocene. We also find that the Eocene–Oligocene transition in south-eastern Tibet witnessed leaf-size diminution and a floral composition change from sub-tropical/warm temperate to cool temperate, likely reflective of both uplift and secular climate change, and that, by the latest Eocene, floral modernization on Tibet had already taken place, implying modernization was deeply rooted in the Palaeogene.

## INTRODUCTION

**Figure 1.**
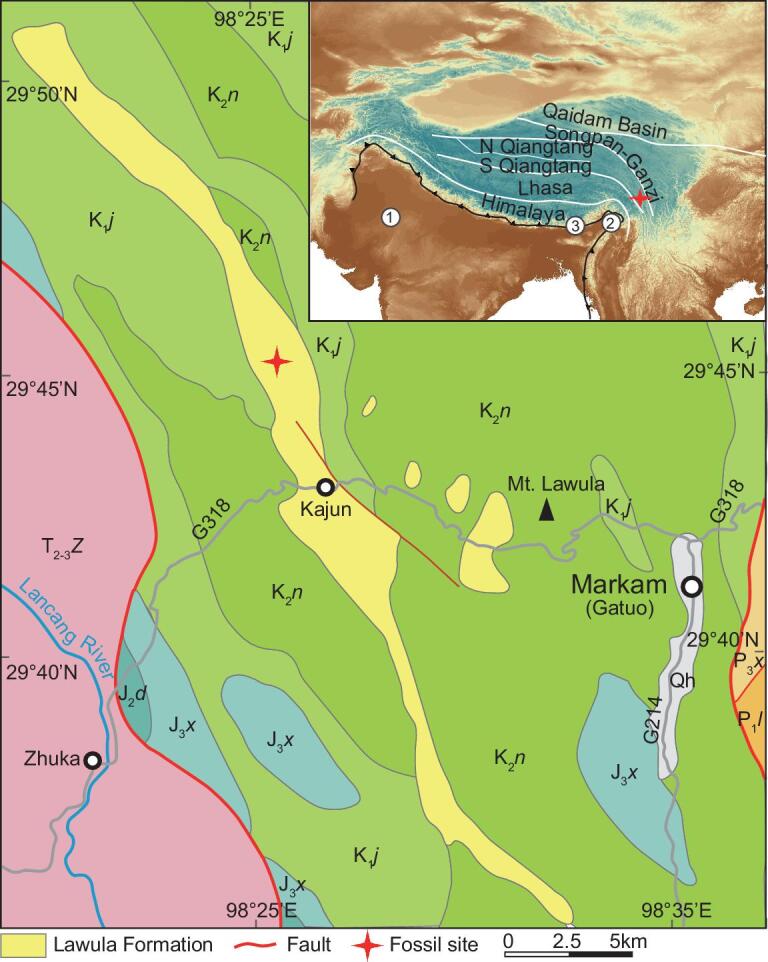
Geological map of the Markam Basin in south-eastern Tibet. The red star shows the position of the Markam Basin. Inset: overview map of the Himalaya-Tibet Edifice showing major geological components. 1–3 are locations of fossil sites used to estimate moist enthalpy at sea level. 1. The early Eocene Gurha assemblage of Rajasthan [42]; 2. The late Oligocene Tirap assemblage, Makum Coalfield, Assam India [41]; 3. The mid-Miocene lower Siwalik succession of the Kameng River, north-eastern India [36]. P_1_*l*, Lower Permian Licha Formation; P_3_*x*, Upper Permian Xiayacun Formation; T_2–3_*Z*, Middle Triassic Zhuka Formation; J_2_*d*, Middle Jurassic Dongdaqiao Formation; J_3_*x*, Upper Jurassic Xiaosuoka Formation; K_1_*j*, Lower Cretaceous Jingxing Formation; K_2_*n*, Upper Cretaceous Nanxin Formation; Qh, Holocene deposits [44].

The Tibetan Plateau today has an average elevation of more than 4.5 km spread over an area of ∼2.5 million km^2^ and, together with the adjacent Himalaya and Hengduan mountain systems (Fig. 1), form the Himalaya-Tibet Edifice (HTE), the most prominent orographic feature on Earth. The HTE has long been considered a major influence on Asian Monsoon atmospheric circulation [1,2]. However, the Tibetan Plateau is not a single geological entity, but a fusion of several continental terranes that accreted to the southern margin of Asia during the Palaeozoic and Mesozoic eras [3,4] and it is becoming clear that a Proto-Tibetan Highland (PTH) existed long before India impacted Eurasia [5,6] and before the rise of the Himalaya [7]. The presence of a high PTH in the Palaeogene challenges numerous molecular phylogenetic studies that link biotic diversification to a Neogene uplift of the Tibetan Plateau [8–10].

This PTH, made up of the Lhasa and Qiangtang terranes (Fig. 1), hosted two major mountain systems. The Lhasa Terrane had a high (∼4.5 km) southern flank at 56 Ma in the form of the Andes-like Gangdese Arc highlands [11] that pre-dated the rise of the Himalaya [7] and was separated from the Qiangtang Terrane uplands (the elevation of which is loosely constrained but may have been >4 km [11]) by an east–west (E–W) trending elongate lowland along the Bangong–Nujiang suture, represented in part today by the Nima and Lunpola basins. Currently, the palaeoelevation histories of the floors of these basins are poorly quantified, but the inferred lowland floor could have persisted below ∼3 km until as recently as the early Miocene [12,13].

Tectonism, expressed as pronounced topographic relief, and biodiversity are intimately linked with many of Earth's biodiversity hotspots located in low-latitude mountainous regions. This includes those of south-eastern Tibet and Yunnan [14]. Despite uncertainties in palaeoelevation determinations, what is apparent is that the Palaeogene PTH cannot be described as a plateau. However, our current understanding of the processes of landscape evolution in the south-eastern part of the HTE has been undermined by recent discoveries that key sedimentary basins used for palaeoelevation determinations and biostratigraphic correlation are much older than previously thought [15,16], leading to a call for more integrated and diverse palaeoaltimetric approaches within well-constrained chronostratigraphic frameworks [17].

Moreover, the elevation history of the Tibetan Plateau is not a simple scaling of the India-Asia convergence increasing over time since the onset of that collision. Instead it is one of collisional modification of a large pre-existing area of high and complex topography [5] that, at low latitudes and with millennial and longer climate fluctuations, likely supported a Palaeogene ‘speciation pump’ [10]. Such a pump could have contributed to the modernization of the Asian biota long before the Neogene. But to explore this requires well-preserved fossils placed within a rigorous absolute dating framework that is independent of biostratigraphy. Here, we use radiometrically dated plant-fossil assemblages in an attempt to quantify when south-eastern Tibet achieved its present elevation, and what kind of floras existed there at that time.

## RESULTS AND DISCUSSION

### The importance of landscape evolution on the south-eastern margin of Tibet

More than 90% of the relative motion between the Indian and Eurasian plates has been absorbed by deformation at the margins of the plateau, while internal shortening of the plateau, including topographic modification of the PTH, accounts for a little over a third of the total convergence [18]. Today, surface deformation, as revealed by global positioning system (GPS) velocities, is low in the interior of the plateau, but high in the Himalaya and along the south-eastern and north-eastern margins of the plateau [19]. North–south normal faulting and dyke development on the Lhasa Terrane are indicative of E–W extension taking place as early as the Eocene [20,21]. While the mechanism driving this extension remains unclear and surface expression of E–W extension in terms of normal faults is minor (≤40 km) [22], it is possible that surface faulting reflects more extensive plastic rock movement at depth (termed lower crustal flow) [23]. One result of this would have been uplift along the south-eastern margin of the Tibetan Plateau, triggering changes in drainage incision [24] and thus increases in close proximity niche diversity that is an essential component of any speciation pump [10]. When this uplift began and what caused it are matters of considerable uncertainty and debate [17].

On the south-eastern edge of the Tibetan Plateau, strike-slip faulting along the Gaoligong and Ailao-Red River fault systems commenced simultaneously at ∼33–32 Ma [25–29], indicating that little or no lower crustal extrusion in south-eastern Tibet occurred before the start of the Oligocene [30]. Since then, however, the ∼1300 ± 410 km of crustal shortening in the northern part of the Qiangtang Terrane appears to have been accommodated in part by extrusion and rotation of the northern Qiangtang Terrane to the south-east [30], suggesting uplift associated with this extrusion should have begun in the Oligocene. Isotope-derived surface-elevation estimates for this part of the Qiangtang Terrane at the start of the extrusion process are now regarded as suspect [15–17] because, in many cases, a Miocene age was assumed based on biostratigraphy. A relatively high (∼3 km) south-eastern Tibet in the early Oligocene or earlier would imply a longer history of surface uplift, possibly involving crustal thickening during the Eocene, leading to south-eastward extrusion of a portion of the Qiangtang Terrane during the Oligocene to early Miocene and, if it exists, lower crustal flow had a far earlier onset than currently envisaged. What is needed is a palaeosurface height measurement that is based on absolute dating and free of the ‘educated guesses’ that underpin isotopic palaeoelevation estimates [17].

To quantify the surface elevation of south-eastern Tibet and characterize a key component of the Asian biota in the Palaeogene, we date radiometrically a succession of plant megafossil assemblages in the Markam (sometimes called Mangkang) Basin—a small pull-apart basin within the Qiangtang Terrane [31] (Fig. 1). We then determine their taxonomic composition and, to measure past surface elevation, use the multivariate statistical proxy (CLAMP, Climate Leaf Analysis Multivariate Program) [32,33] to derive moist enthalpy values encoded in fossil-leaf form for both the Markam Basin and similarly aged sea-level palaeofloras. CLAMP quantitatively interprets a range of palaeotemperature and moisture variables (Table 1) from the morphology of fossil leaves (details on the CLAMP website: http://clamp.ibcas.ac.cn). Moist enthalpy is particularly well coded in fossil woody dicot leaf form [34] and palaeo-moist enthalpy derived from fossil leaves using CLAMP has been used to determine past surface elevations for south central Tibet [35,36] in the mid-Miocene as well as the uplift of the Himalaya [7]. Put simply, but subject to palaeospatial correction (see the ‘Methods’ section), the difference between sea-level moist enthalpy (*H*_sea level_) and that of a site of similar age at an unknown elevation (e.g. a Tibetan site—*H*_Tibet_) divided by the gravitational acceleration constant (*g*) yields the difference in height (Z) between sea level and the elevated site [37]:

(1) \begin{eqnarray*} Z = \left( {{H_{{\rm{sea}}\,{\rm{level}}}}-{H_{{\rm{Tibet}}}}} \right)/g. \end{eqnarray*}

**Table 1.** Results of CLAMP analyses of fossil-leaf assemblages and predicted palaeoelevations. MAAT, mean annual air temperature; WMMAT, warm-month mean air temperature; CMMAT, cold-month mean air temperature; LGS, length of the growing season; GSP, growing season precipitation; MMGSP, mean monthly growing season; 3WET, precipitation during three consecutive wettest months; 3DRY, precipitation during the three consecutive driest months; RH, relative humidity; SH, specific humidity; ENTH, moist enthalpy; ENTHPC, moist enthalpy corrected to the end Eocene Mangkang palaeoposition; ELEVR, elevation using moist enthalpy at sea level not adjusted to the Mangkang palaeoposition; ELEVA, elevation with position adjusted moist enthalpy at sea level. Uncertainties are ±1 standard deviation.

Assemblage	MAAT	WMMAT	CMMAT	LGS	GSP	MMGSP	3WET	3DRY	RH	SH	ENTH	ENTHPC	ELEVR	ELEVA
	(°C)	(°C)	(°C)	(Month)	(mm)	(mm)	(mm)	(mm)	(%)	(g/kg)	(kJ/kg)	(kJ/kg)	(km)	(km)
MK1	16.4	28	3.2	9.7	1704	179	768	196	64	7.9	320.9	N/A	3.59	3.9
MK3	17.8	28	4.8	10.3	2157	234	957	313	72	10	330.2	N/A	2.64	2.9
Gurha72	23.9	27.9	18.9	12	1792	153	931	106	78	14.1	351.8	357.6	0	0
Gurha39	24.7	28.2	19	12	1838	158	984	83	79	14.5	353.8	359.6	0	0
Tirap	26.2	28.4	20	12	2126	192	1110	97	81	15.1	356.9	358.4	0	0
Kameng R	25.3	27.8	21.3	12	1741	140	962	73	81	14.9	355.8	N/A	0	0
Uncertainties	± 2.3	± 2.8	± 3.6	± 1.1	± 606	± 61	± 358	± 95	± 8.4	± 1.9	± 0.9	± 1.1	± 1.6	± 0.9

### The geology and palaeofloras of the Markam Basin, south-eastern Tibet

Four distinct plant-fossil assemblages (MK1–4) were collected by members of the Palaeoecology Group, Xishuangbanna Tropical Botanical Garden, from the Lawula Formation (Markam Basin) exposed near Kajun village (29.7527° N, 98.4327° E), ∼16 km north-west of the town of Gatuo, Mangkang County, south-eastern Tibet (Fig. 1). Today, the site is at an elevation of 3910 m with a mean annual air temperature (MAAT) of 4.1°C and mean annual precipitation of 618 mm.

**Figure 2.**
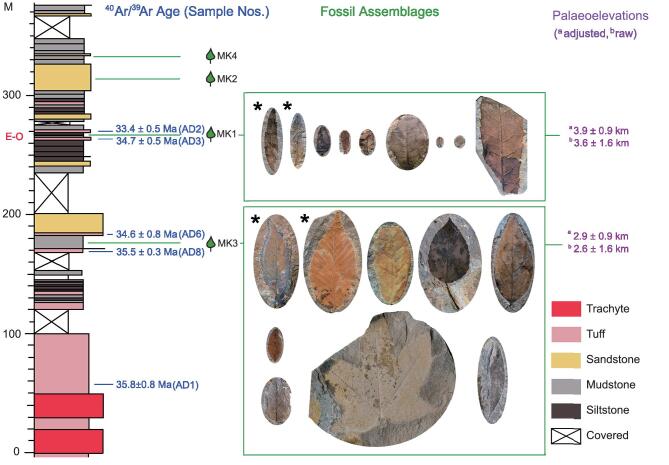
Markam measured section in the Lawula Formation. ^40^Ar/^39^Ar sample locations and dates constrain the ages of the MK1 and MK3 leaf assemblages, for which indicative selected leaf fossils are shown to scale, together with predicted palaeoelevations. A distinct reduction in leaf size is evident between MK3 and MK1, which is situated at the onset of the Eocene–Oligocene transition (E–O). Adjusted elevations are where moist enthalpy at sea level obtained from Indian fossil floras have been transposed to the palaeoposition of the Markam Basin. The most abundant taxa in terms of specimens recovered are marked with an asterisk (*).

The oldest units in the Markam Basin, assigned to the Lower Cretaceous (Jingxing Fm.), are predominantly red in colour and consist of calcareous quartz sandstones, calcareous siltstones and mudstones with gypsum interbeds. In the middle part of the succession, Eocene–Oligocene conglomerates, sandstones and high-K volcanic rocks are overlain unconformably by the fossiliferous Lawula Fm., which consists of interbedded sandstones and mudstones [38] and abundant high-K volcaniclastics [39] (Fig. 2).

Our fossil leaves are preserved as abundant impressions in mudstones, siltstones and sandstones associated with thin carbonaceous beds and palaeosols. Overall, the succession represents river channel, pond, lake and swamp environments typical of a floodplain, but with episodic influxes of volcanic ash and debris flows (Fig. 2).

Assemblage MK3 has so far yielded over 2634 specimens divisible into four conifer species and 36 evergreen and deciduous woody dicot leaf morphotypes (species) preserved in a buff to grey siltstone (Supplementary Figs 1 and 2, available as Supplementary Data at *NSR* online). The assemblage is dominated by evergreen round cupule oaks (*Quercus* subg. *Cyclobalanopsis*) with lesser quantities of members of the Betulaceae (*Alnus* and *Betula*) [40]. Conifers are represented by *Pinus*, *Chamaecyparis*, *Tsuga* and *Abies* (Supplementary Fig. 1, available as Supplementary Data at *NSR* online).

The MK1 sediment package overlies a succession of carbonaceous mudstones and thin coals and has so far yielded 692 specimens representing 24 woody dicot morphotypes illustrated in Fig. 2 and Supplementary Fig. 3, available as Supplementary Data at *NSR* online. This assemblage is dominated by *Salix*, while *Rosa*, alpine oaks and *Alnus* are fewer in number. Conifers are represented by *Picea*.

Some 35 m higher in the section, the MK2 assemblage is made up of fragmented and poorly preserved leaves of *Alnus*, *Betula*, alpine oak (*Quercus* section *Heterobalanus*) and *Rhododendron*, occurring within a ∼20 m-thick fluvial fine to medium sandstone. Immediately overlying this sandstone, a mudstone yields assemblage MK4, which consists solely of abundant fruits and pollen of *Hemitrapa*, an aquatic angiosperm similar to modern *Trapa*, which inhabits quiet, shallow, pond environments. These assemblages are illustrated in Supplementary Fig. 4, available as Supplementary Data at *NSR* online.

The lack of obligate thermophyllic taxa and the dominance of evergreen oaks in assemblage MK3 are in stark contrast to the distinctly tropical sea-level Cenozoic floras typical across northern India [41,42] and qualitatively point to assemblage MK3 representing upland vegetation. Moreover, the loss of the MK3 evergreen oak *Cyclobalanopsis* from MK1 and MK2 in favour of deciduous taxa better adapted to cold conditions may suggest ongoing cooling and/or yet higher elevations for these assemblages.

The preservation of MK3 leaves is good and, using associated reproductive organs, we were able to assign many specimens to living genera. However, preservational limitations of the MK1 assemblage meant that many of these leaves could not be assigned reliably to living genera and mostly could only be divided into morphotypes but, because CLAMP does not rely on taxonomic assignment, we are still able to obtain climate and elevation estimates, albeit with some limitations.

### Dating framework

The Lawula Formation in the Kajun village area was previously considered to be late Miocene based on floristic comparison [43] and lithostratigraphy [44], although potassic volcanic rocks attributable to the Lawula Fm. elsewhere in the basin have been dated as 33.5 ± 0.2 Ma [39]. Previous surface height estimates assumed a Miocene age [45]. We collected our dating samples from volcanic rocks immediately above and below the fossiliferous MK1 and MK3 horizons (Fig. 2) and determined their ages using single crystal laser ablation ^40^Ar/^39^Ar analysis (see ‘Methods’ and Supplementary Table 1, available as Supplementary Data at *NSR* online, for analytical details). The bottom part of the section was deposited at 35.6 ± 0.8 Ma, while the tuff overlying the MK1 assemblage is dated as 33.4 ± 0.5 Ma. Taking into account uncertainties, the likely maximum period of deposition encompassing these two leaf beds is 3.5 myrs, while the minimum is 0.9 myrs.

The four leaf-bearing horizons are notably different in composition. The oldest assemblage, MK3, is of an evergreen and deciduous mixed sub-tropical-to-warm temperate type and is underlain and overlain by tuffs dated as 35.5 ± 0.3 and 34.61 ± 0.8 Ma, respectively, making the likely age of the assemblage latest Eocene and very close to the Eocene–Oligocene boundary, which is currently dated at 33.9 Ma [46].

The diminutive leaves of assemblage MK1 have a distinctly more stressed and temperate aspect than MK3 and occur ∼90 m stratigraphically above MK3 in a grey siltstone bounded below and above by water-lain ash horizons dated as 34.7 ± 0.5 and 33.4 ± 0.5 Ma, respectively (Fig. 2). This places the assemblage at the onset of the Eocene–Oligocene (E–O) cooling event [47], making assemblages MK2 and MK4 early Oligocene in age. The change in floristic composition from sub-tropical to temperate is consistent with cooling across the E–O transition [47], but could also be due to surface uplift.

### Climate at the Eocene-Oligocene transition in south-eastern Tibet

Only assemblages MK3 and MK1 are well enough preserved and suitably diverse to allow a palaeoclimate determination. To do this, we employ the well-established non-taxonomic CLAMP proxy (see the ‘Methods’ section) to derive palaeotemperature and precipitation regimes. The architecture (physiognomy) of woody dicot fossil leaves retains a record of the environment to which they were exposed in life and, on a global scale, climate determines leaf form more strongly than taxonomic affiliation [48]. In the context of Asia, leaf form appears particularly suited to the detection and characterization of ancient monsoon regimes [49].

Using CLAMP, we find that MK3 assemblage yields a MAAT of 17.8 ± 2.3°C, with a warm-month mean air temperature (WMMAT) of 28.1 ± 2.8°C and a cold-month mean air temperature (CMMAT) of 4.8 ± 3.6°C (Table 1). As suggested by the taxonomic composition, this is warmer than the overlying MK1 assemblage (MAAT 16.4 ± 2.8°C) but the difference is small and within uncertainty. Both the WMMAT and CMMAT derived from MK1 are also cooler than MK3 but overlap within uncertainty. More noticeable is a reduction of nearly 80% in the growing season precipitation accompanied by an increase in rainfall seasonality. Assemblage MK3 has a wet/dry season precipitation ratio of 3:1 whereas, for MK1, it is a little over 4:1, with MK1 having a much drier dry season.

MK3 and MK1 exhibit a marked difference in leaf size as well as species composition (Fig. 2). Small leaves can lead to CLAMP yielding anomalously warm temperatures (the ‘alpine nest’ effect [33]) so we interpret the MK1 environment as being cooler than the CLAMP values suggest.

The cooling observed between MK3 and MK1 could be due to two factors: (i) MK3 is latest Eocene and MK1 is earliest Oligocene and the cooling reflects the onset of the global drop in marine temperatures observed across this interval [47] or (ii) the cooling reflects uplift of the Markam area within the time represented by the 90 m or so of sediment deposition that separates the two assemblages. A possible third scenario represents a combination of secular climate cooling and uplift.

### Estimating the palaeoelevations of the plant-fossil assemblages

**Figure 3.**
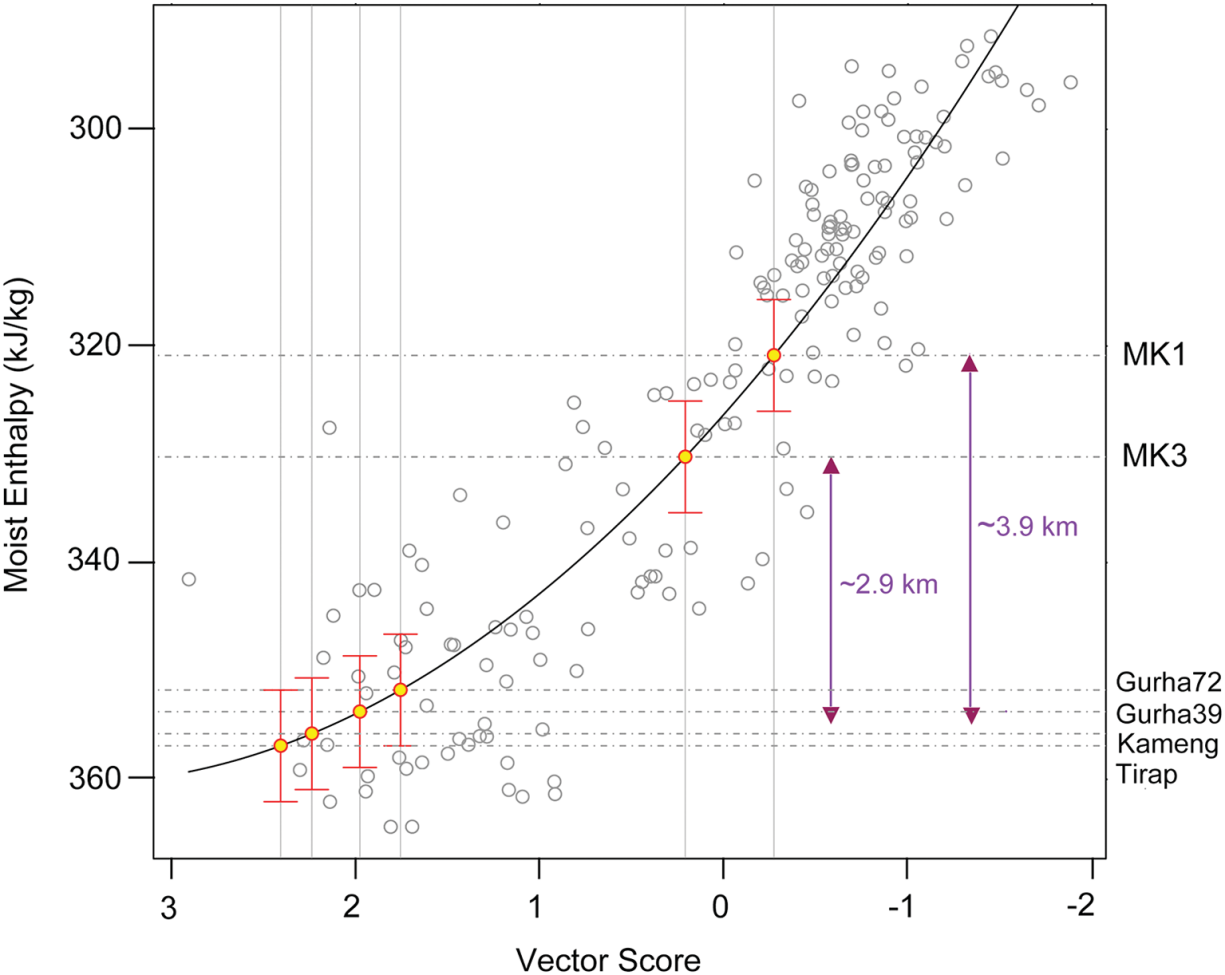
CLAMP enthalpy regression. Plots using the PhysgAsia2 and high-resolution gridded climate data calibration show the positions of the MK1 and MK3 assemblages and those at palaeo-sea level (Gurha39 and Gurha72 (early Eocene), Tirap (late Oligocene) and Kameng River (middle Miocene), northern India). Bars show 1 SD uncertainty. The vector score is a dimensionless number indicating the position along the moist enthalpy trend through physiognomic space defined by 177 modern vegetation sites growing under quantified climate ‘normals’ spanning 30 years (1961–90) primarily from across Asia and North America. See the ‘Methods’ section for further details.

Leaf form in woody dicotyledonous flowering plants is remarkably good at encoding moist enthalpy—a property of the atmosphere related to altitude [37] (Fig. 3). To obtain palaeoelevations of the MK1 and MK3 assemblages, we need to know the palaeo-moist enthalpy of the atmosphere surrounding them, and the moist enthalpy encoded in coeval fossil-leaf assemblages known to represent vegetation growing very near sea level. Although reliably dated co-eval sea-level floras are unknown in India, regional moist enthalpy at sea level (MESL) appears to have remained within narrow limits from Eocene to mid-Miocene times (Fig. 3), allowing us to use averaged/interpolated values from previously published Palaeogene sea-level floras.

The MK3 leaf assemblage yields a moist enthalpy value of 330.2 ± 1.1 kJ/kg, while the MK1 assemblage translates to 320.9 ± 1.1 kJ/kg (Fig. 3). At the time they were formed, the sea-level assemblages were further south than the Markam site and, because moist enthalpy tends to be zonal, adjustments to the raw MESL values have to be made to estimate MESL for the palaeo-location of the Markam Basin. We did this using the same climate model-derived spatial MESL fields as in previous studies [7,36]. Using the temporally interpolated early Eocene to late Oligocene palaeolatitude-adjusted MESL of 358.7 ± 1 kJ/kg, the relationship expressed in Equation (1) yields an absolute elevation a.m.s.l. of 2.91 ± 0.91 and 3.85 ± 0.91 km for Mk3 and MK1, respectively. Assuming no secular climate change between the two assemblages, this translates to a maximum rise in elevation of ∼1 km within the 0.9–3.5 million years separating the two leaf deposits, and that the Markam area attained near its present elevation in the early Oligocene. Uncertainties (Table 1) include both sea-level MESL variation and statistic uncertainty inherent in the CLAMP proxy. The raw elevation values (uncorrected for palaeoposition) are ∼300 m lower. A simple arithmetic mean of the Palaeogene MESLs yields almost identical elevations. However, the reduced diversity of the MK1 assemblage means that the MK1 enthalpy values, and hence surface height determination, should be treated with caution. Combined with the current uncertainty regarding the degree of secular climate change experienced at the Markam site, this means that the absolute elevation of the MK1 assemblage is poorly constrained, but the enthalpy difference between MK3 and MK1 could be indicative of a rapidly rising landscape at the E–O transition.

## SUMMARY AND IMPLICATIONS

Our finding that the surface elevation of south-eastern Tibet was ∼3 km and rising to close to its present height at the E–O transition demonstrates clearly the early onset of uplift in this region, rather than later during the late Oligocene and Neogene [24]. Note that our elevation measurements do not suffer from diagenetic alteration or on assumptions regarding time/topography-dependent isotopic lapse rates, so can contribute to the future refinement of isotope-based palaeoaltimetric proxies as recently called for [17]. Our findings, employing well-dated fossil floras and using a technique independent of isotope fractionation models and lapse rates, show that the elevation of south-eastern Tibet largely took place prior to the Oligocene, which has major implications for uplift mechanisms, landscape development and biotic evolution, countering arguments for a Neogene onset of lower crustal flow, uplift and river incision [24].

Our ^40^Ar/^39^Ar analysis of the volcanic ashes bounding the Markam fossil floras as latest Eocene to earliest Oligocene, and not Miocene as previously reported [43], adds to a growing list of sites in south-eastern Tibet and Yunnan where radiometric dating has shown them to be far older than previously thought based on biostratigraphy and lithostratigraphy [15,16,50]. Many fossil biotas of modern composition were previously regarded as Miocene or younger in age, but this assumed that the modernization of the biotas was a Neogene phenomenon. Now that non-biological dating methods have shown this assumption to be invalid in several sedimentary basins on the south-eastern margin of Tibet, our ideas about the evolution of both biotas and landscape require substantial revision, but it is already clear that the evolution of the modern highly diverse Asian biota is a Palaeogene, not a Neogene, phenomenon and took place before the E–O transition and so is unrelated to it. This implies a modernization, possibly driven by the HTE ‘speciation pump’, deeply rooted in the Palaeogene.

## METHODS

### Geochronology

Samples for ^40^Ar/^39^Ar dating were analysed at the Open University, UK. The samples were crushed using a pestle and mortar, and the crushate was sieved and washed repeatedly in de-ionized water to remove dust and clay particles from the surfaces of all the size fractions. Using a binocular microscope, feldspar/biotite crystals were picked, selecting pieces free from alteration. The samples were cleaned ultrasonically in acetone and de-ionized water, dried using a hot plate and packaged in aluminium foil packets of ∼10 × 10 mm in size prior to irradiation.

Samples were irradiated at the McMaster Nuclear Reactor (McMaster University, Canada) for 94 hours. Cadmium shielding was used and the samples were held in position 8E. Neutron flux was monitored using biotite mineral standard GA1550, which has an age of 98.5 ± 0.5 Ma [51]. Standards were packed for irradiation, either side of the unknown samples, and analysed using the single-grain fusion method using a 1059-nm CSI fibre laser and a MAP215–50 mass spectrometer. The J-values were then calculated by linear extrapolation between the two measured J-values and a 0.5% error on J was used.

The samples were analysed using a MAP 215–50 mass spectrometer, which was operated by LabVIEW software. The irradiated samples were loaded into an ultra-high vacuum system and mounted on a New Wave Research UP-213 stage and a 1059-nm CSI fibre laser was focused into the sample chamber and was used to melt the sample. Extracted gases were cleaned for 5 minutes using two SAES AP-10 getters running at 450°C and room temperature. System blanks were measured before every two sample analyses. Gas clean-up and inlet were fully automated, with measurement of ^40^Ar, ^39^Ar, ^38^Ar, ^37^Ar and ^36^Ar, each for 10 scans, and the final measurements are extrapolations back to the inlet time.

Data reduction utilized ArArCALC v2.5.2 [52]. The system blanks measured before every two sample analyses were subtracted from the raw sample data. All data were corrected for mass spectrometer discrimination using values of 283. Results were corrected ^37^Ar decay since irradiation and for neutron-induced interference reactions, using the default correction factors in ArArCALC: (^40^Ar/^36^Ar)_trapped_ = 295.5, (^40^Ar/^36^Ar)_cosmogenic_ = 0.018 ± 35%, (^38^Ar/^36^Ar)_trapped_ = 0.1869, (^38^Ar/^36^Ar)_cosmogenic_ = 1.493 ± 3%, (^39^Ar/^37^Ar)_Ca_ = 0.000699, (^36^Ar/^37^Ar)_Ca_ = 0.00027, (^40^Ar/^39^Ar)_K_ = 0.01024, K/Ca = 0.57. Weighted average of ages were also calculated using Isoplot version 3.7 [53]. Further details of the analyses are given as Supplementary Table 1, available as Supplementary Data at *NSR* online.

### Fossil material

Plant fossils were excavated from surface exposures, numbered, cleaned and photographed using a Nikon D800 camera and natural light, and are archived in the collections at Xishuangbanna Tropical Botanical Garden, CAS.

Numerical descriptions of leaf forms followed the normal CLAMP protocols (http://clamp.ibcas.ac.cn) based on 36 morphotypes (species) from MK3 (Supplementary Figs 1 and 2, available as Supplementary Data at *NSR* online) and 24 morphotypes from MK1 (Supplementary Fig. 3, available as Supplementary Data at *NSR* online). CLAMP Scoresheets numerically describing leaf forms are given in Supplementary Table 2, available as Supplementary Data at *NSR* online.

### CLAMP analysis

All climate and elevation data were obtained from numerically scored woody dicot leaf forms using the CLAMP [32,33,54] calibrated using the PhysgAsia2 leaf-form training set and its accompanying high-resolution gridded climate data [55] (GRIDMetPhysgAsia2). This calibration set has been validated in the Tibet region against multiple isotope systems [56,57] in locations where simple Rayleigh distillation applies.

The small leaf size evident in the MK1 assemblage is similar to that seen in cold locations and within CLAMP physiognomic space (termed the ‘alpine nest’) [33] can give anomalously warm climate estimates. MK1 appears to represent a stressed flora and plots outside PhysgAsia2 calibration space. This introduces unquantifiable uncertainties in the CLAMP environmental estimates and the MK1 elevation estimates can only be regarded as indicative.

### Phytopalaeoaltimetry

Moist enthalpy is particularly well archived in leaf form [37,58] and that obtained from fossil leaves has been used to derive palaeoelevations in North America [58], Tibet [35,36] and the Himalaya [7].

Moist static energy (*h*), the total specific energy content of air, is given by:

(2) \begin{eqnarray*} h = c'_pT + L_vq + gZ, \end{eqnarray*}

where *c′_p_* the specific heat capacity at a constant pressure of moist air, *T* is the temperature (in K), *L_v_* is the latent heat of vapourization of water, *q* is the specific humidity, *g* is the acceleration due to gravity (a constant), and *Z* is elevation. Note that *h* is conserved as an air parcel rises and passes over a topographic barrier [37]. Moist static energy is made up of two components: enthalpy and potential energy:

(3) \begin{eqnarray*} h = H + gZ, \end{eqnarray*}

where *H* is enthalpy (*c′pT* + *Lvq*) and *gZ* is potential energy. As a parcel of air rises, it gains potential energy and, because moist static energy remains the same, enthalpy decreases.

It follows therefore that, because the value of *h* is conserved, the difference in elevation between two locations at the same latitude is given by:

(4) \begin{eqnarray*} \Delta Z = \left( {{H_{low}} - {H_{high}}} \right)/g. \end{eqnarray*}

Convection means that *h* remains more or less the same throughout the troposphere [59,60] so, by deriving *H* from fossil-leaf form using CLAMP, the differences in elevation between two fossil-leaf assemblages can be determined.

MESL was obtained using fossil-leaf archives recovered and previously published from the Tirap open-cast mine in the Makum Coalfield, Assam India (27.2888°N, 95.77083°E) dated as late Oligocene (∼23 Ma) [41], the early Eocene (∼56 Ma) Gurha assemblages of Rajasthan (27.87398°N, 72.86709°E) [42] and the middle Miocene (13 Ma) Kameng River assemblages, eastern Siwaliks, India [36]. Despite a timespan of over 40 million years, MESL values only range from 353 to 357 kJ/kg. This limited variation in MESL values over time removes the need for precise age congruence [7]. We use the arithmetic mean and temporally interpolated MESL values of the Palaeogene (Gurha and Tirap) floras to estimate the absolute surface height of the Markam floras and relative elevational change across the E–O transition.

Because moist enthalpy tends to vary across latitudes, we made corrections for palaeospatial trends in MESL derived from general circulation palaeoclimate models using a previously published methodology that employed the same Indian fossil archives [7,36]. Sea-surface moist enthalpy fields were generated using a numerical climate model with Eocene and Miocene boundary conditions (http://www.bridge.bris.ac.uk), with interpolation for the Oligocene. Differences in MESL between the palaeolatitude of the Indian and Tibetan sites are used to obtain MESL at the position of the Tibetan site. The Markam Basin is situated on the southernmost tip of the extruded Qiangtang Terrane and the exact palaeolatitude of the Markam floras at the E–O transition is poorly constrained but likely was between ∼35 °N [61] and ∼22 °N [30]. We chose 29 °N as our Markam reference palaeolatitude. When corrections are made for palaeoposition, temporal variation in enthalpy reduce considerably (Table 1), resulting in no more than 300 m of elevation change, but with improved precision.

## Supplementary Material

nwy062_Supplemental_Files
