# Supplementary material for: Uplift, climate and biotic changes at the Eocene–Oligocene transition in south-eastern Tibet
Source: Natl Sci Rev. 2018 Jun 12;6(3):495–504. doi: 10.1093/nsr/nwy062 (PMC8291530; doi:10.1093/nsr/nwy062)
Supplement: nwy062_Supplemental_Files [file nwy062_supplemental_files.zip › Supplementary Table 1.pdf]

Ar/Ar ages in stratigraphical order

	Average Age	Inverse Isochron Age
AD2	33.4±0.5	32.4±2.0
AD3	34.7±0.5	34.9±0.8
AD6	34.6±0.8	33.0±2.0
AD8	35.5±0.3	34.3±1.0
AD1	35.8±0.8	36.6±1.2

J-Values used for analysis

	J-value	Error of J-value	Error of J-value
AD2	0.0064443	0.0000497	0.77%
AD3	0.006456	0.0000491	0.76%
AD6	0.0064911	0.0000475	0.73%
AD8	0.0065146	0.0000463	0.71%
AD1	0.0064325	0.0000503	0.78%

AD2

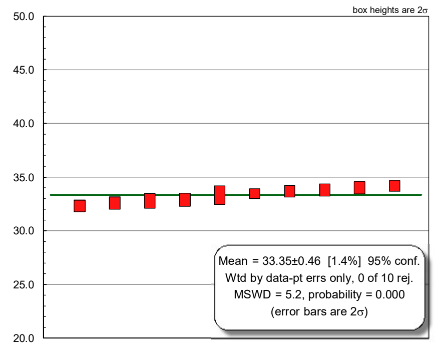

Inverse Isochron			39(k)/40(a+r)	± 2σ	36(a)/40(a+r)	± 2σ	r.i.
W16A52180	4 °C	4	0.3216680	± 0.0047716	0.00017747	± 0.00002655	0.0066
W16A52181	4 °C	4	0.3367815	± 0.0052664	0.00009584	± 0.00006740	0.0052
W16A52183	4 °C	4	0.3370350	± 0.0049023	0.00004637	± 0.00002726	0.0054
W16A52184	4 °C	4	0.3482512	± 0.0050971	0.00005721	± 0.00003267	0.0029
W16A52186	4 °C	4	0.3347234	± 0.0050630	0.00007929	± 0.00001906	0.0093
W16A52187	4 °C	4	0.3493239	± 0.0047084	0.00007625	± 0.00003386	0.0023
W16A52189	4 °C	4	0.3395809	± 0.0045963	0.00011048	± 0.00003939	0.0102
W16A52190	4 °C	4	0.3437962	± 0.0061507	0.00008094	± 0.00003442	0.0170
W16A52195	4 °C		0.3234536	± 0.0059530	0.00001525	± 0.00002592	0.0025
W16A52196	4 °C	4	0.3282076	± 0.0042912	0.00009610	± 0.00002318	0.0069
W16A52197	4 °C	4	0.3295674	± 0.0039550	0.00015098	± 0.00001893	0.0098

Results		40(a)/36(a)	± 2σ	40(r)/39(k)	± 2σ		Age (Ma)	± 2σ	MSWD
Inverse Isochron<br>Error Chron		587.36	± 292.20<br>± 49.75%	2.80543	± 0.09274<br>± 3.31%		32.40	± 1.17<br>± 3.61%	3.28<br>0%
						Full External Error		± 1.33	
						Analytical Error		± 1.06	

Statistics				
	2σ Confidence Limit	2.00	Convergence	0.0017451572
	Error Magnification	1.8118	Number of Iterations	4
	Number of Data Points	10	Calculated Line	Weighted York-2
	Spreading Factor	7.8%		

Relative Abundances			36Ar [V]	%1σ	37Ar [V]	%1σ	38Ar [V]	%1σ	39Ar [V]	%1σ	40Ar [V]	%1σ
W16A52180	4 °C	4	0.0003591	7.347	0.0170520	9.042	0.0078603	4.627	0.6393783	0.717	1.994206	0.190
W16A52181	4 °C	4	0.0000682	33.842	0.0071418	20.284	0.0028155	6.527	0.2306554	0.685	0.687229	0.376
W16A52183	4 °C	4	0.0001120	27.155	0.0246170	9.970	0.0085478	3.574	0.7523065	0.643	2.239784	0.338
W16A52184	4 °C	4	0.0001148	26.401	0.0258083	13.069	0.0074359	3.850	0.6462757	0.689	1.862341	0.247
W16A52186	4 °C	4	0.0003193	11.609	0.0276479	9.499	0.0152658	3.755	1.3025533	0.698	3.904712	0.290
W16A52187	4 °C	4	0.0001881	21.313	0.0203256	11.026	0.0093478	2.984	0.8271603	0.648	2.376318	0.185
W16A52189	4 °C	4	0.0001799	17.365	0.0123044	17.153	0.0057873	7.399	0.5388408	0.579	1.592274	0.349
W16A52190	4 °C	4	0.0001402	20.783	0.0063316	35.121	0.0063265	3.311	0.5824614	0.691	1.700157	0.566
W16A52195	4 °C		0.0000204	69.896	0.0097766	16.113	0.0042814	6.707	0.3561191	0.808	1.104615	0.439
W16A52196	4 °C	4	0.0002809	11.680	0.0233845	9.991	0.0107243	2.398	0.9295074	0.611	2.841539	0.232
W16A52197	4 °C	4	0.0005465	6.148	0.0259708	11.607	0.0135913	2.733	1.1709631	0.568	3.564966	0.191
		Σ	0.0023293	4.354	0.2003607	3.873	0.0919838	1.221	7.9762214	0.213	23.868142	0.092

Relative Abundances	40(r)/39(k)	± 2σ	Age (Ma)	± 2σ	40Ar(r) (%)	39Ar(k) (%)	K/Ca	± 2σ
W16A52180	2.94576	± 0.05018	34.01	± 0.57	94.44	8.02	21.4	± 3.9
W16A52181	2.88519	± 0.07457	33.31	± 0.85	96.83	2.89	18.4	± 7.5
W16A52183	2.92640	± 0.04893	33.78	± 0.56	98.29	9.43	17.4	± 3.5
W16A52184	2.82294	± 0.04982	32.60	± 0.57	97.96	8.10	14.3	± 3.7
W16A52186	2.91754	± 0.04738	33.68	± 0.54	97.32	16.33	26.9	± 5.1
W16A52187	2.79817	± 0.04741	32.32	± 0.54	97.40	10.37	23.2	± 5.1
W16A52189	2.84867	± 0.05185	32.90	± 0.59	96.40	6.76	25.0	± 8.6
W16A52190	2.83913	± 0.05921	32.79	± 0.68	97.27	7.30	52.4	± 36.8
W16A52195	3.07770	± 0.06145	35.51	± 0.70	99.22	4.46	20.8	± 6.7
W16A52196	2.96033	± 0.04410	34.17	± 0.50	96.83	11.65	22.7	± 4.5
W16A52197	2.89890	± 0.03886	33.47	± 0.44	95.22	14.68	25.7	± 6.0

AD3

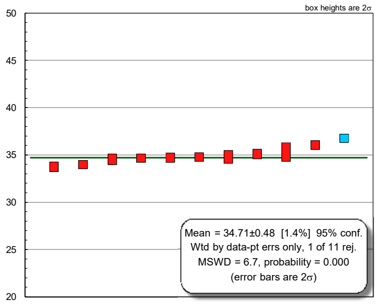

Inverse Isochron			39(k)/40(a+r)	± 2σ	36(a)/40(a+r)	± 2σ	r.i.
W16A52220	4 °C	4	0.3232165	± 0.0052484	0.00009400	± 0.00003477	0.0084
W16A52221	4 °C	4	0.3273016	± 0.0040376	0.00005468	± 0.00001667	0.0062
W16A52225	4 °C	4	0.3385267	± 0.0041618	0.00001879	± 0.00001894	0.0020
W16A52226	4 °C	4	0.3264459	± 0.0043230	0.00003175	± 0.00002096	0.0052
W16A52228	4 °C	4	0.3199892	± 0.0042105	0.00022515	± 0.00002330	0.0391
W16A52229	4 °C	4	0.3024111	± 0.0060722	0.00026036	± 0.00006263	0.0088
W16A52233	4 °C	4	0.3093255	± 0.0035856	0.00005509	± 0.00000927	0.0053
W16A52234	4 °C	4	0.3257856	± 0.0044689	0.00007487	± 0.00001544	0.0217
W16A52236	4 °C	4	0.3294659	± 0.0036014	0.00005605	± 0.00003937	0.0019
W16A52237	4 °C	4	0.3292561	± 0.0037050	0.00004593	± 0.00001953	0.0017
W16A52239	4 °C	4	0.3121658	± 0.0036798	0.00009136	± 0.00002005	0.0019

Results	40(a)/36(a)	± 2σ	40(r)/39(k)	± 2σ	Age (Ma)	± 2σ	MSW
Inverse Isochron<br>Error Chron	309.56	± 183.78<br>± 59.37%	3.02629	± 0.08480<br>± 2.80%	34.99	± 1.10<br>± 3.16%	15.79<br>0%
					Full External Error	± 1.30	
					Analytical Error	± 0.97	

Statistics				
2σ Confidence Limit	1.94	Convergence	0.0002301860
Error Magnification	3.9731	Number of Iterations	8
Number of Data Points	11	Calculated Line	Weighted York-2
Spreading Factor	10.9%		

Relative Abundances			36Ar [V]	%1σ	37Ar [V]	%1σ	38Ar [V]	%1σ	39Ar [V]	%1σ	40Ar [V]	%1σ
W16A52220	4 °C	4	0.0001759	17.606	0.0255803	10.530	0.0063124	4.657	0.576004	0.730	1.787942	0.355
W16A52221	4 °C	4	0.0003001	14.432	0.0418442	8.161	0.0196780	1.787	1.701667	0.568	5.216415	0.240
W16A52225	4 °C	4	0.0000561	42.851	0.0230255	13.406	0.0092806	3.189	0.859403	0.561	2.547409	0.250
W16A52226	4 °C	4	0.0001053	30.632	0.0182488	15.259	0.0113965	1.653	1.005564	0.570	3.090595	0.335
W16A52228	4 °C	4	0.0008028	5.087	0.0320986	6.006	0.0126470	3.870	1.124516	0.547	3.525677	0.364
W16A52229	4 °C	4	0.0002428	11.882	0.0075956	14.836	0.0033486	4.397	0.278724	0.949	0.924508	0.326
W16A52233	4 °C	4	0.0004069	8.036	0.0439350	6.622	0.0254722	1.858	2.184432	0.557	7.084188	0.160
W16A52234	4 °C	4	0.0002646	9.938	0.0233857	8.067	0.0128517	2.356	1.110625	0.563	3.420390	0.391
W16A52236	4 °C	4	0.0001599	33.740	0.0146864	8.622	0.0098859	4.262	0.902918	0.512	2.749767	0.190
W16A52237	4 °C	4	0.0002286	19.235	0.0656246	6.155	0.0169502	3.216	1.483447	0.545	4.520502	0.140
W16A52239	4 °C	4	0.0005554	10.709	0.0309128	7.595	0.0210917	2.832	1.852390	0.579	5.952894	0.109
		Σ	0.0032982	3.964	0.3269375	2.678	0.1489146	0.886	13.079690	0.187	40.820288	0.075

Relative Abundances	40(r)/39(k)	± 2σ	Age (Ma)	± 2σ	40Ar(r) (%)	39Ar(k) (%)	K/Ca	± 2σ
W16A52220	3.00796	± 0.05850	34.78	± 0.67	96.90	4.40	12.8	± 2.7
W16A52221	3.00592	± 0.04010	34.76	± 0.46	98.06	13.01	23.2	± 3.8
W16A52225	2.93757	± 0.03975	33.97	± 0.46	99.10	6.57	21.3	± 5.7
W16A52226	3.03456	± 0.04453	35.08	± 0.51	98.73	7.69	31.4	± 9.6
W16A52228	2.91718	± 0.04473	33.74	± 0.51	93.04	8.60	20.0	± 2.4
W16A52229	3.05234	± 0.08699	35.29	± 1.00	92.02	2.13	20.9	± 6.2
W16A52233	3.18021	± 0.03796	36.75	± 0.43	98.06	16.70	28.3	± 3.8
W16A52234	3.00159	± 0.04378	34.71	± 0.50	97.46	8.49	27.1	± 4.4
W16A52236	2.98495	± 0.04812	34.52	± 0.55	98.01	6.90	35.0	± 6.1
W16A52237	2.99593	± 0.03802	34.64	± 0.44	98.31	11.34	12.9	± 1.6
W16A52239	3.11695	± 0.04139	36.03	± 0.47	96.99	14.16	34.2	± 5.2

AD6

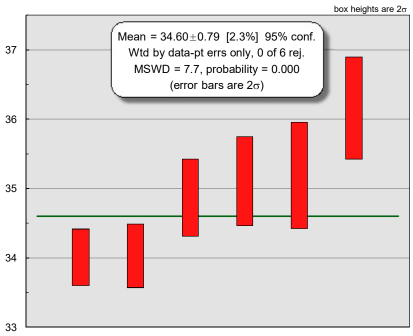

Inverse Isochron			39(k)/40(a+r) ± 2σ	36(a)/40(a+r) ± 2σ	r.i.
W16A52081	4 °C	4	0.1954022 ± 0.0025441	0.00051312 ± 0.00005425	0.0082
W16A52086	4 °C	4	0.2857208 ± 0.0039776	0.00037522 ± 0.00004432	0.0120
W16A52088	4 °C	4	0.2318798 ± 0.0043944	0.00057537 ± 0.00005083	0.0583
W16A52089	4 °C		0.0448531 ± 0.0007835	0.00065326 ± 0.00009064	0.0182
W16A52091	4 °C	4	0.3369240 ± 0.0040817	0.00004515 ± 0.00001938	0.0006
W16A52092	4 °C	4	0.2796347 ± 0.0033274	0.00051853 ± 0.00005156	0.0096
W16A52094	4 °C	4	0.2356804 ± 0.0031842	0.00082845 ± 0.00006494	0.0184
W16A52095	4 °C	4	0.3338980 ± 0.0037324	0.00007701 ± 0.00001377	0.0027
W16A52097	4 °C	4	0.3245773 ± 0.0046591	0.00008835 ± 0.00002307	0.0021
W16A52098	4 °C	4	0.2482317 ± 0.0033526	0.00030705 ± 0.00003493	0.0108
W16A52100	4 °C	4	0.3009170 ± 0.0036174	0.00030761 ± 0.00004181	0.0160

Results	40(a)/36(a) ± 2σ		40(r)/39(k) ± 2σ		Age ± 2σ (Ma)		MSWD
Inverse Isochron<br>Error Chron	646.66	± 211.86<br>± 32.76%	2.83942	± 0.18141<br>± 6.39%	33.03	± 2.14<br>± 6.49%	35.40<br>0%
					Full External Error	± 2.24	
					Analytical Error	± 2.09	

Statistics				
	2σ Confidence Limit	2.00	Convergence	0.0003166834
	Error Magnification	5.9502	Number of Iterations	4
	Number of Data Points	10	Calculated Line	Weighted York-2
	Spreading Factor	40.2%		

Relative Abundances			36Ar [V]	%1σ	37Ar [V]	%1σ	38Ar [V]	%1σ	39Ar [V]	%1σ	40Ar [V]	%1σ
W16A52081	4 °C	4	0.0016623	5.270	0.0102522	14.548	0.0075229	2.566	0.6313951	0.629	3.237688	0.168
W16A52086	4 °C	4	0.0008513	5.853	0.0261056	7.788	0.0075349	5.242	0.6428653	0.660	2.256497	0.222
W16A52088	4 °C	4	0.0003802	4.379	0.0016430	48.270	0.0019751	8.521	0.1528780	0.809	0.660859	0.493
W16A52089	4 °C		0.0010458	6.917	0.0063519	17.941	0.0012006	5.823	0.0716787	0.808	1.598712	0.332
W16A52091	4 °C	4	0.0001586	20.585	0.0144084	10.374	0.0125019	3.686	1.1353345	0.599	3.381300	0.090
W16A52092	4 °C	4	0.0011390	4.944	0.0158560	8.878	0.0074106	3.070	0.6111132	0.571	2.191616	0.168
W16A52094	4 °C	4	0.0016029	3.905	0.0081214	16.208	0.0057726	6.974	0.4550492	0.638	1.935425	0.220
W16A52095	4 °C	4	0.0007010	8.731	0.0338828	9.097	0.0342248	2.005	2.9692504	0.547	8.923020	0.115
W16A52097	4 °C	4	0.0002707	12.820	0.0090842	14.231	0.0116286	2.421	0.9767108	0.704	3.019159	0.141
W16A52098	4 °C	4	0.0006419	5.658	0.0060142	18.592	0.0059911	3.528	0.5166241	0.644	2.086490	0.203
W16A52100	4 °C	4	0.0010100	6.731	0.0239223	10.232	0.0116766	2.294	0.9793527	0.544	3.264533	0.255
		Σ	0.0077610	2.401	0.1034308	5.524	0.0923699	1.243	7.8565214	0.271	28.042307	0.066

Relative Abundances	40(r)/39(k) ± 2σ	Age ± 2σ (Ma)	40Ar(r) (%)	39Ar(k) (%)	K/Ca ± 2σ
W16A52081	4.34168 ± 0.10001	50.26 ± 1.14	84.67	8.04	35.1 ± 10.2
W16A52086	3.11186 ± 0.06345	36.16 ± 0.73	88.65	8.18	14.0 ± 2.2
W16A52088	3.57935 ± 0.09649	41.53 ± 1.11	82.80	1.95	53.0 ± 51.2
W16A52089	17.99119 ± 0.67983	199.73 ± 7.15	80.66	0.91	6.4 ± 2.3
W16A52091	2.92843 ± 0.03935	34.05 ± 0.45	98.33	14.45	44.9 ± 9.3
W16A52092	3.02814 ± 0.06561	35.20 ± 0.76	84.44	7.78	22.0 ± 3.9
W16A52094	3.20431 ± 0.09292	37.23 ± 1.07	75.34	5.79	31.9 ± 10.4
W16A52095	2.92678 ± 0.03494	34.03 ± 0.40	97.39	37.79	50.0 ± 9.1
W16A52097	3.00050 ± 0.04796	34.88 ± 0.55	97.07	12.43	61.3 ± 17.5
W16A52098	3.66298 ± 0.06497	42.49 ± 0.74	90.70	6.58	49.0 ± 18.2
W16A52100	3.02110 ± 0.05525	35.12 ± 0.64	90.63	12.47	23.3 ± 4.8

AD8

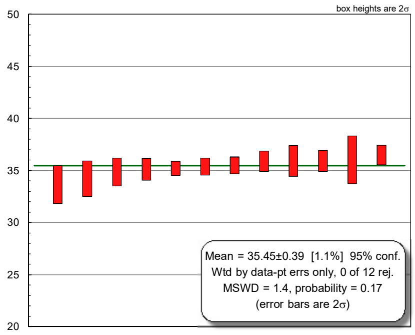

Inverse Isochron			39(k)/40(a+r) ± 2σ	36(a)/40(a+r) ± 2σ	r.i.
W16A51991	4 °C	4	0.2846553 ± 0.0081655	0.00040936 ± 0.00016959	0.0048
W16A51992	4 °C	4	0.2878014 ± 0.0043330	0.00038558 ± 0.00007075	0.0055
W16A51994	4 °C	4	0.3065107 ± 0.0048368	0.00025605 ± 0.00003641	0.0035
W16A51995	4 °C	4	0.2820792 ± 0.0038938	0.00044754 ± 0.00011415	0.0055
W16A51997	4 °C	4	0.3159322 ± 0.0057660	0.00030319 ± 0.00015847	0.0068
W16A52038	4 °C	4	0.2780489 ± 0.0046479	0.00049033 ± 0.00006390	0.0247
W16A52039	4 °C	4	0.2597994 ± 0.0037227	0.00063407 ± 0.00005754	0.0263
W16A52041	4 °C	4	0.2997509 ± 0.0051692	0.00029976 ± 0.00004648	0.0142
W16A52042	4 °C	4	0.3099832 ± 0.0070562	0.00025229 ± 0.00009630	0.0118
W16A52044	4 °C	4	0.3048004 ± 0.0063236	0.00028154 ± 0.00006478	0.0184
W16A52045	4 °C	4	0.3060111 ± 0.0044800	0.00024563 ± 0.00005573	0.0067
W16A52047	4 °C	4	0.3097156 ± 0.0064394	0.00031199 ± 0.00013942	0.0128

Results	40(a)/36(a) ± 2σ		40(r)/39(k) ± 2σ		Age ± 2σ (Ma)		MSWD
Inverse Isochron	377.67	± 59.03	2.94120	± 0.07541	34.32	± 1.00	0.42
		± 15.63%		± 2.56%		± 2.90%	94%
					Full External Error	± 1.20	
					Analytical Error	± 0.87	

Statistics				
	2σ Confidence Limit	1.89	Convergence	0.0001365816
	Error Magnification	1.0000	Number of Iterations	4
	Number of Data Points	12	Calculated Line	Weighted York-2
	Spreading Factor	16.5%		

Relative Abundances			36Ar [V]	%1σ	37Ar [V]	%1σ	38Ar [V]	%1σ	39Ar [V]	%1σ	40Ar [V]	%1σ	40(r)/39(k) ± 2σ	Age ± 2σ (Ma)	40Ar(r) (%)	39Ar(k) (%)	K/Ca ± 2σ
W16A51991	4 °C	4	0.0001721	20.629	0.0012645	50.778	0.0017314	3.798	0.1191905	1.384	0.419936	0.375	3.08806 ± 0.19746	36.02 ± 2.28	87.65	3.17	53.7 ± 54.6
W16A51992	4 °C	4	0.0004984	9.135	0.0040560	15.617	0.0050299	3.078	0.3705007	0.727	1.291133	0.194	3.07872 ± 0.08638	35.91 ± 1.00	88.35	9.84	52.1 ± 16.3
W16A51994	4 °C	4	0.0004751	7.004	0.0200868	8.528	0.0076067	3.727	0.5605255	0.777	1.834424	0.139	3.01567 ± 0.05923	35.18 ± 0.68	92.14	14.89	15.9 ± 2.7
W16A51995	4 °C	4	0.0003395	12.717	0.0016947	31.984	0.0024845	4.686	0.2133957	0.654	0.758691	0.219	3.07628 ± 0.12712	35.88 ± 1.47	86.53	5.67	71.8 ± 45.9
W16A51997	4 °C	4	0.0001744	25.869	0.0046837	21.487	0.0025082	5.564	0.1799619	0.820	0.571454	0.400	2.88165 ± 0.15761	33.63 ± 1.82	90.75	4.78	21.9 ± 9.4
W16A52038	4 °C	4	0.0008783	6.488	0.0038818	23.348	0.0066000	3.467	0.4967068	0.751	1.791477	0.366	3.07538 ± 0.08618	35.87 ± 1.00	85.27	13.19	72.9 ± 34.1
W16A52039	4 °C	4	0.0009650	4.521	0.0018812	48.271	0.0051260	3.525	0.3947606	0.654	1.523519	0.292	3.12792 ± 0.08029	36.48 ± 0.93	81.05	10.49	119.6 ± 115.5
W16A52041	4 °C	4	0.0003731	7.704	0.0035796	32.426	0.0053436	6.762	0.3710058	0.805	1.241504	0.307	3.04059 ± 0.07012	35.47 ± 0.81	90.86	9.86	59.1 ± 38.3
W16A52042	4 °C	4	0.0001258	18.854	0.0040049	22.409	0.0019387	7.733	0.1527661	1.019	0.494376	0.505	2.98548 ± 0.11486	34.83 ± 1.33	92.25	4.06	21.7 ± 9.8
W16A52044	4 °C	4	0.0001706	11.395	0.0035583	22.098	0.0026167	8.129	0.1830487	0.925	0.602419	0.467	3.00788 ± 0.08935	35.09 ± 1.03	91.40	4.86	29.3 ± 13.0
W16A52045	4 °C	4	0.0004625	11.218	0.0132448	11.653	0.0072283	5.081	0.5699718	0.693	1.868392	0.236	3.03066 ± 0.06998	35.35 ± 0.81	92.45	15.14	24.5 ± 5.7
W16A52047	4 °C	4	0.0001544	22.269	0.0002855	190.416	0.0019319	3.861	0.1528040	0.885	0.494933	0.544	2.93110 ± 0.14702	34.20 ± 1.70	90.49	4.06	305.1 ± 1162.0
		Σ	0.0047891	2.888	0.0622217	5.602	0.0501461	1.500	3.7646380	0.245	12.892258	0.092					

AD1

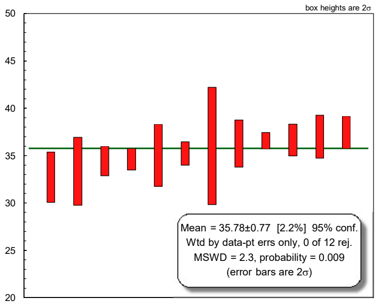

Inverse Isochron			39(k)/40(a+r) ± 2σ		36(a)/40(a+r) ± 2σ		r.i.
W16A51972	4 °C	4	0.2740126	± 0.0099548	0.00047950	± 0.00048783	0.0037
W16A51973	4 °C	4	0.3255633	± 0.0107891	0.00018729	± 0.00032578	0.0061
W16A51975	4 °C	4	0.3266012	± 0.0135912	0.00023875	± 0.00021812	0.0073
W16A51976	4 °C	4	0.2967745	± 0.0075608	0.00018401	± 0.00012079	0.0087
W16A51978	4 °C	4	0.2837650	± 0.0060450	0.00035402	± 0.00019671	0.0080
W16A51979	4 °C	4	0.3187045	± 0.0135341	0.00009798	± 0.00027207	0.0051
W16A52016	4 °C	4	0.3019585	± 0.0051097	0.00009641	± 0.00019455	0.0008
W16A52017	4 °C	4	0.3004562	± 0.0066300	0.00007301	± 0.00012884	0.0031
W16A52019	4 °C	4	0.2954633	± 0.0047176	0.00020330	± 0.00005753	0.0101
W16A52020	4 °C	4	0.2928904	± 0.0052892	0.00034904	± 0.00009167	0.0071
W16A52022	4 °C	4	0.2898660	± 0.0062523	0.00044924	± 0.00011608	0.0135
W16A52023	4 °C	4	0.2751681	± 0.0046181	0.00057985	± 0.00007683	0.0427

Results	40(a)/36(a) ± 2σ		40(r)/39(k) ± 2σ		Age ± 2σ (Ma)		MSWD
Inverse Isochron<br>Error Chron	231.58	± 88.76<br>± 38.33%	3.17415	± 0.10093<br>± 3.18%	36.55	± 1.15<br>± 3.15%	2.17<br>2%
					Full External Error	± 1.35	
					Analytical Error	± 1.15	

Statistics				
	2σ Confidence Limit	1.89	Convergence	0.0005490319
	Error Magnification	1.4722	Number of Iterations	3
	Number of Data Points	12	Calculated Line	Weighted York-2
	Spreading Factor	16.7%		

Relative Abundances			36Ar [V]	%1σ	37Ar [V]	%1σ	38Ar [V]	%1σ	39Ar [V]	%1σ	40Ar [V]	%1σ	40(r)/39(k) ± 2σ		Age ± 2σ (Ma)		40Ar(r) (%)	39Ar(k) (%)	K/Ca ± 2σ	
W16A51972	4 °C	4	0.0000519	50.771	0.0000218	2147.148	0.0005360	11.064	0.0296171	1.720	0.1083901	0.582	3.13237	± 0.53866	36.07	± 6.14	85.59	2.16	775.1	± 33285.8
W16A51973	4 °C	4	0.0000290	82.182	0.0054239	14.761	0.0006927	15.331	0.0477037	1.367	0.1470036	0.933	2.90161	± 0.31149	33.44	± 3.56	94.15	3.48	5.0	± 1.5
W16A51975	4 °C	4	0.0000313	45.175	0.0008265	66.546	0.0006059	13.858	0.0423752	1.906	0.1301783	0.832	2.84582	± 0.23090	32.80	± 2.64	92.64	3.09	29.2	± 38.9
W16A51976	4 °C	4	0.0000799	32.528	0.0012413	47.323	0.0018950	10.446	0.1278059	1.122	0.4319557	0.602	3.18634	± 0.14569	36.69	± 1.66	94.28	9.33	58.7	± 55.6
W16A51978	4 °C	4	0.0001009	27.691	0.0002210	192.984	0.0013406	5.900	0.0806182	0.948	0.2849269	0.484	3.15539	± 0.21610	36.34	± 2.46	89.28	5.89	207.9	± 802.4
W16A51979	4 °C	4	0.0000152	137.615	0.0001366	406.240	0.0008816	9.799	0.0491435	1.736	0.1547011	1.219	3.04685	± 0.28409	35.10	± 3.24	96.79	3.59	205.1	± 1666.3
W16A52016	4 °C	4	0.0000275	98.396	0.0017443	49.063	0.0010433	8.252	0.0840275	0.806	0.2791314	0.256	3.21736	± 0.19806	37.04	± 2.26	96.85	6.14	27.5	± 26.9
W16A52017	4 °C	4	0.0000225	86.871	0.0001172	522.332	0.0015770	6.744	0.0911643	0.956	0.3043529	0.549	3.25647	± 0.14587	37.49	± 1.66	97.54	6.66	443.3	± 4630.7
W16A52019	4 °C	4	0.0002349	14.011	0.0042704	16.670	0.0054407	2.352	0.3380694	0.723	1.1476528	0.336	3.18119	± 0.07714	36.63	± 0.88	93.71	24.69	45.1	± 15.1
W16A52020	4 °C	4	0.0001681	13.086	0.0005475	113.802	0.0020097	6.286	0.1406143	0.855	0.4815305	0.289	3.06210	± 0.10809	35.27	± 1.23	89.42	10.27	146.4	± 333.2
W16A52022	4 °C	4	0.0002475	12.864	0.0017012	24.094	0.0024616	5.839	0.1591110	0.987	0.5505373	0.432	2.99190	± 0.13556	34.47	± 1.55	86.47	11.62	53.3	± 25.7
W16A52023	4 °C	4	0.0003781	6.596	0.0001464	388.024	0.0030124	3.146	0.1791049	0.683	0.6527265	0.486	3.01144	± 0.09858	34.69	± 1.12	82.63	13.08	697.4	± 5411.9
		Σ	0.0013869	6.318	0.0160815	13.177	0.0214965	1.834	1.3693552	0.304	4.6730872	0.154								
