# Supplementary material for: Uplift, climate and biotic changes at the Eocene–Oligocene transition in south-eastern Tibet
Source: Natl Sci Rev. 2018 Jun 12;6(3):495–504. doi: 10.1093/nsr/nwy062 (PMC8291530; doi:10.1093/nsr/nwy062)
Supplement: nwy062_Supplemental_Files [file nwy062_supplemental_files.zip › Supplementary Table 2.docx]

**Supplementary Table 2** CLAMP scores of leaf fossil physiognomy in MK1 and MK3.

| Leaf character | | MK3 | MK1 |
| --- | --- | --- | --- |
| Lamina Dissection | Lobed | 0.06 | 0.04 |
| Margin Character States | No Teeth | 0.53 | 0.34 |
|  | Tth Regular | 0.36 | 0.57 |
|  | Teeth Close | 0.17 | 0.23 |
|  | Teeth Round | 0.19 | 0.22 |
|  | Teeth Acute | 0.28 | 0.44 |
|  | Tth Compound | 0.10 | 0 |
| Size Character States | Nanophyll | 0 | 0 |
|  | Leptophyll I | 0 | 0.03 |
|  | Leptophyll II | 0.03 | 0.29 |
|  | Microphyll I | 0.12 | 0.35 |
|  | Microphyll II | 0.44 | 0.22 |
|  | Microphyll III | 0.37 | 0.11 |
|  | Mesophyll I | 0.04 | 0 |
|  | Mesophyll II | 0 | 0 |
|  | Mesophyll III | 0 | 0 |
| Apex Character States | Apex Emarg. | 0 | 0 |
|  | Apex Round | 0.17 | 0.16 |
|  | Apex Acute | 0.20 | 0.61 |
|  | Apex Attenuate | 0.64 | 0.23 |
| Base Character States | Base Cordate | 0.07 | 0.01 |
|  | Base Round | 0.40 | 0.23 |
|  | Base Acute | 0.53 | 0.75 |
| Length to Width Character States | L:W<1:1 | 0.03 | 0 |
|  | L:W 1-2:1 | 0.31 | 0.11 |
|  | L:W 2-3:1 | 0.33 | 0.41 |
|  | L:W 3-4:1 | 0.18 | 0.20 |
|  | L:W>4:1 | 0.15 | 0.28 |
| Shape Character States | Obovate | 0.03 | 0.04 |
|  | Elliptic | 0.83 | 0.83 |
|  | Ovate | 0.14 | 0.13 |
