# Supplementary figures and images for: Uplift, climate and biotic changes at the Eocene–Oligocene transition in south-eastern Tibet
Source: Natl Sci Rev. 2018 Jun 12;6(3):495–504. doi: 10.1093/nsr/nwy062 (PMC8291530; doi:10.1093/nsr/nwy062)

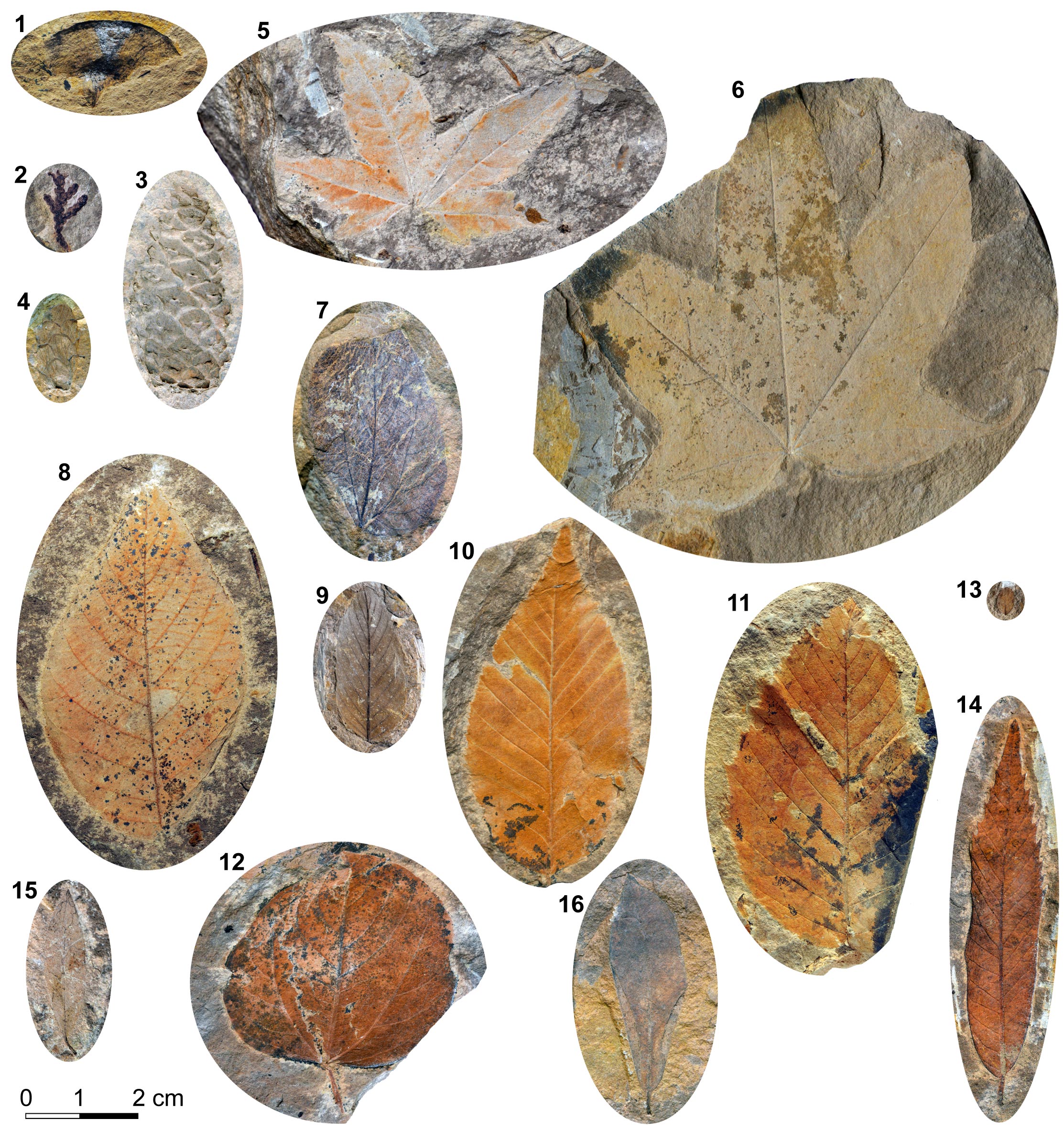

Supplement: nwy062_Supplemental_Files [file nwy062_supplemental_files.zip › Supplementary Figure 1.jpg]

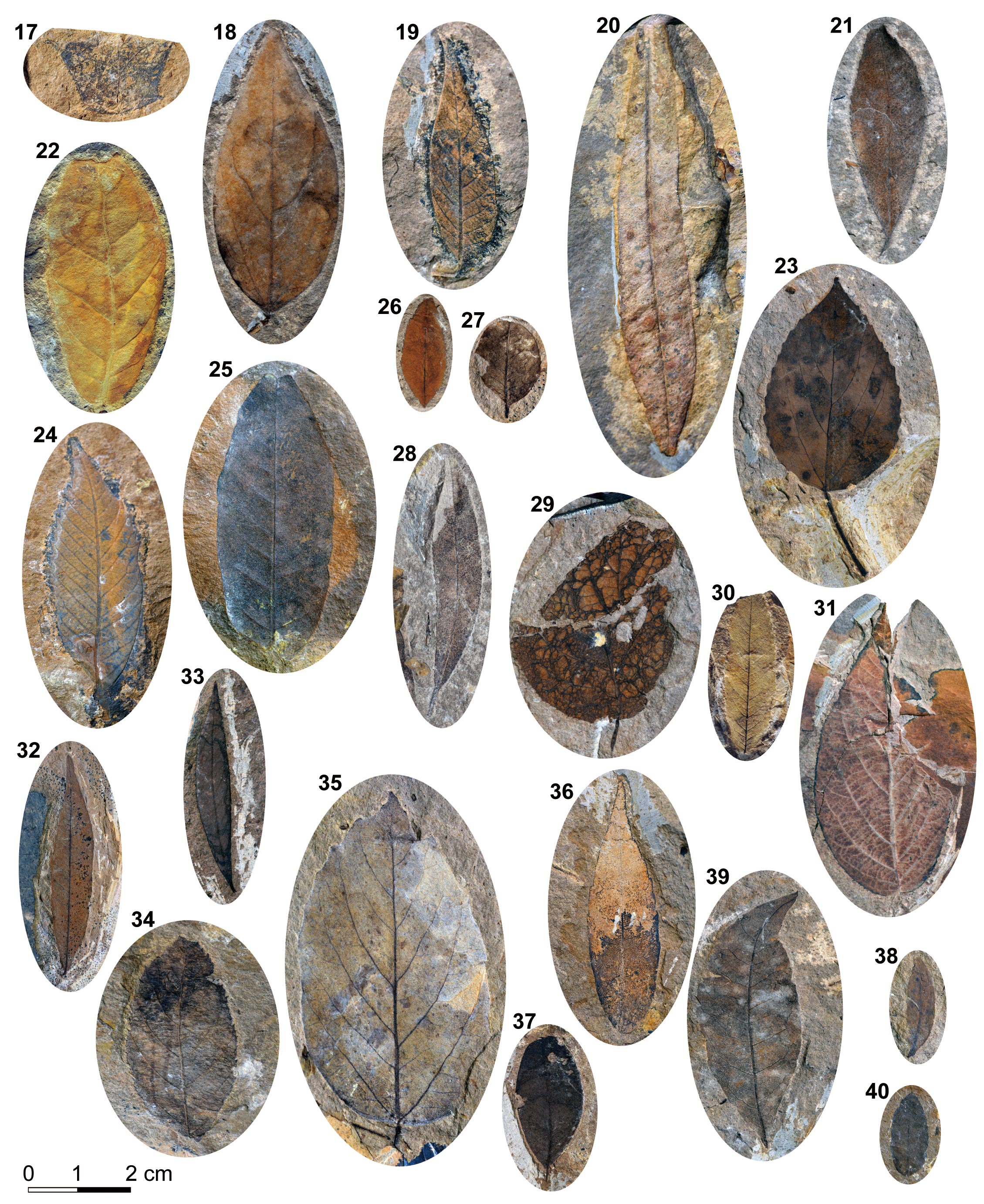

Supplement: nwy062_Supplemental_Files [file nwy062_supplemental_files.zip › Supplementary Figure 2.jpg]

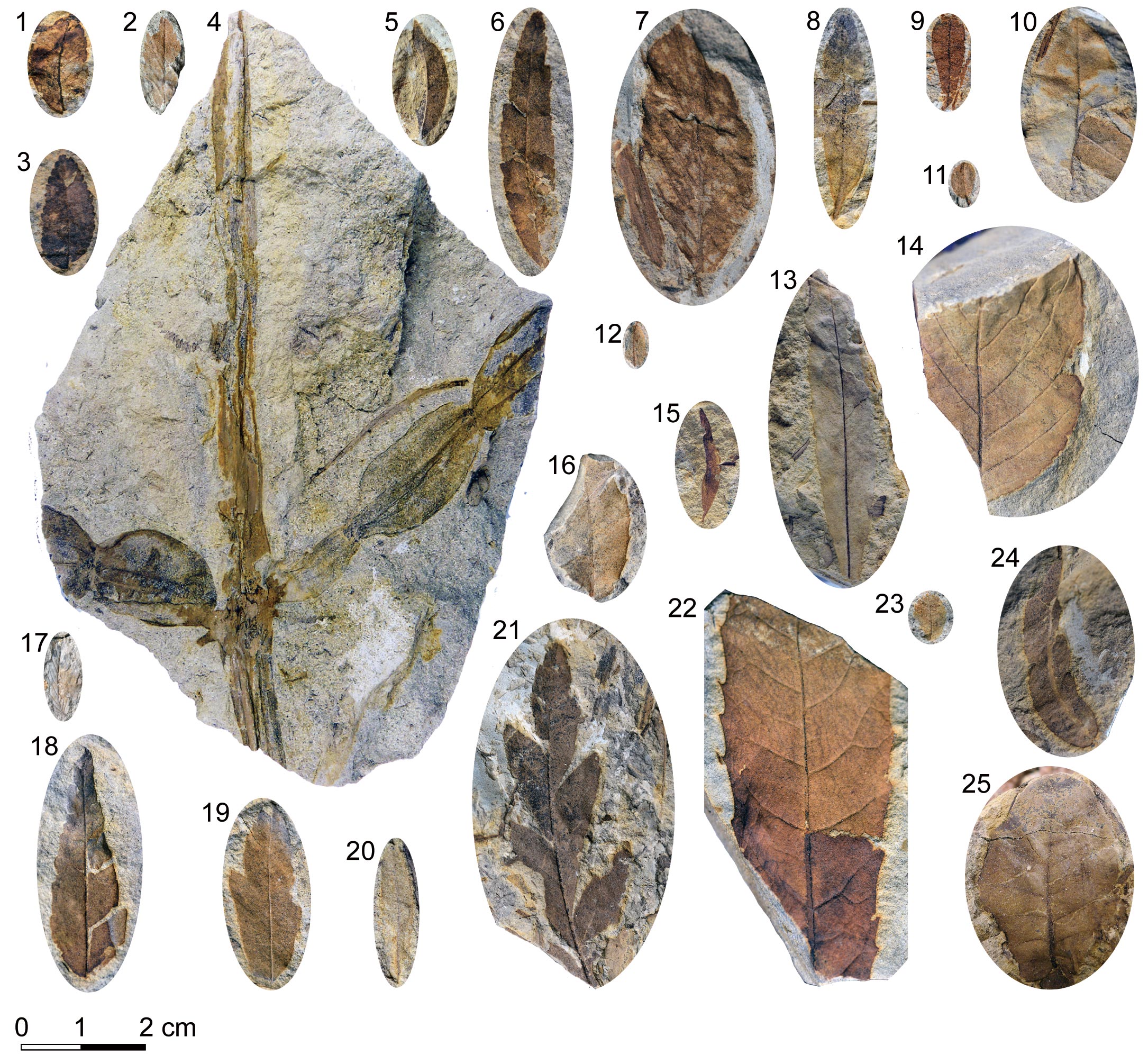

Supplement: nwy062_Supplemental_Files [file nwy062_supplemental_files.zip › Supplementary Figure 3.jpg]

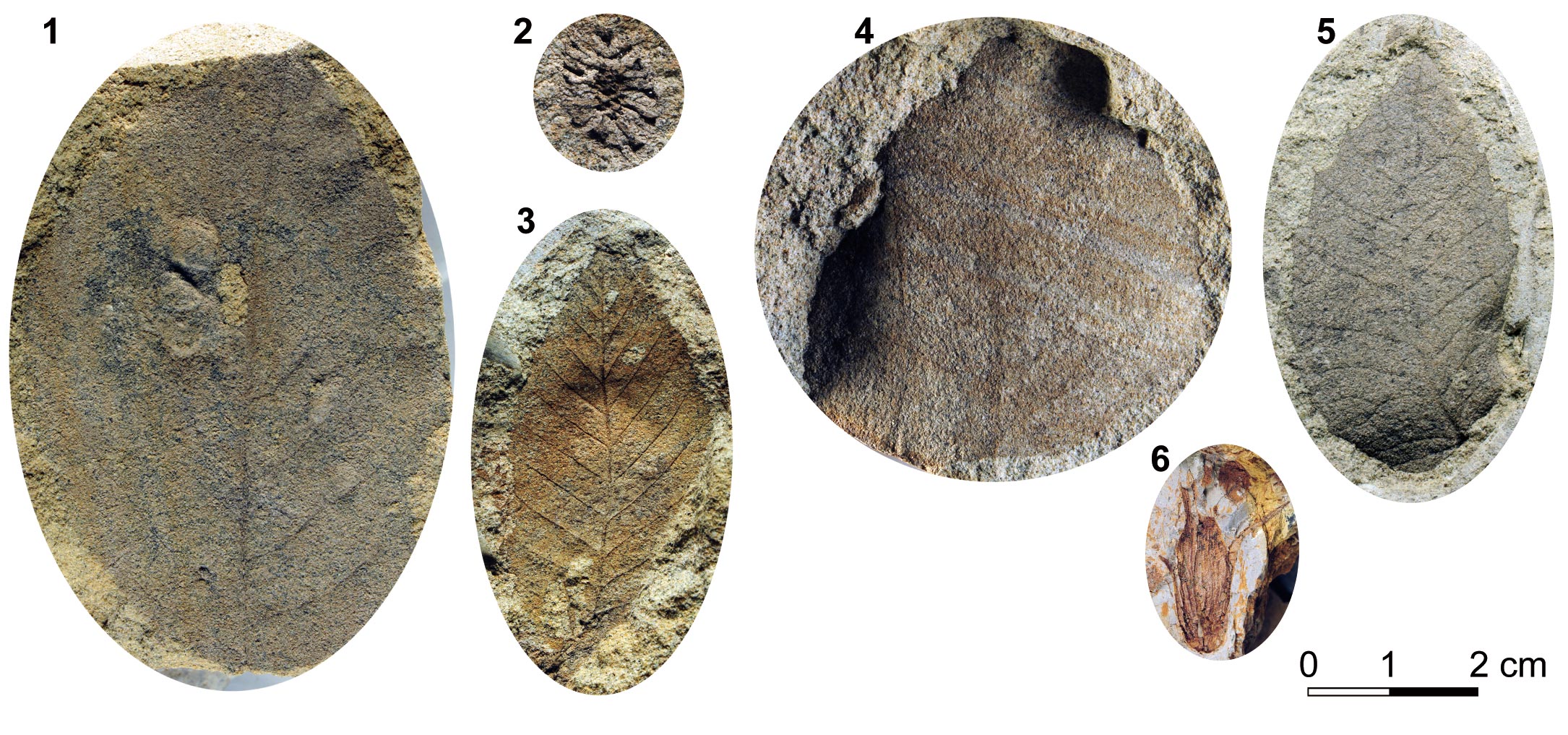

Supplement: nwy062_Supplemental_Files [file nwy062_supplemental_files.zip › Supplementary Figure 4.jpg]
